# Role of FFAR3 in ketone body regulated glucagon-like peptide 1 secretion

**DOI:** 10.1016/j.bbrep.2024.101749

**Published:** 2024-06-07

**Authors:** Sara MT. Persson, Anna Casselbrant, Aiham Alarai, Erik Elebring, Lars Fändriks, Ville Wallenius

**Affiliations:** a Department of Surgery, Institute of Clinical Sciences, Sahlgrenska Academy at the University of Gothenburg, Gothenburg, Sweden; b Department of Surgery, Region Västra Götaland, Sahlgrenska University Hospital, Gothenburg, Sweden; c Department of Surgery, Region Västra Götaland, Sahlgrenska University Hospital Östra, Gothenburg, Sweden

**Keywords:** Free fatty acid receptors, Glucagon-like peptide 1, Incretins, Intestinal ketogenesis, Ketone bodies

## Abstract

**Background:**

Roux-en-Y gastric bypass (RYGB) is an effective treatment for obesity, resulting in long-term weight loss and rapid remission of type 2 diabetes mellitus. Improved glucagon-like peptide 1 (GLP-1) levels is one factor that contributes to the positive effects. Prior to RYGB, GLP-1 response is blunted which can be attributed to intestinal ketogenesis. Intestinal produced ketone bodies inhibit GLP-1 secretion in enteroendocrine cells via an unidentified G-protein coupled receptors (GPCRs). A possible class of GPCRs through which ketone bodies may reach are the free fatty acid receptors (FFARs) located at the basolateral membrane of enteroendocrine cells.

**Aim:**

To evaluate FFAR3 expression in enteroendocrine cells of the small intestine under different circumstances, such as diet and bariatric surgery, as well as explore the link between ketone bodies and GLP-1 secretion.

**Materials and methods:**

FFAR3 and enteroendocrine cell expression was analyzed using Western blot and immunohistochemistry in biopsies from healthy volunteers, obese patients undergoing RYGB and mice. GLUTag cells were used to study GLP-1 secretion and FFAR3 signaling pathways.

**Results:**

The expression of FFAR3 is markedly influenced by diet, especially high fat diet, which increased FFAR3 protein expression. Lack of substrate such as free fatty acids in the alimentary limb after RYGB, downregulate FFAR3 expression. The number of enteroendocrine cells was affected by diet in the normal weight individuals but not in the subjects with obesity. In GLUTag cells, we show that the ketone bodies exert its blocking effect on GLP-1 secretion via the FFAR3, and the Gα_i/o_ signaling pathway.

**Conclusion:**

Our findings that ketone bodies via FFAR3 inhibits GLP-1 secretion bring important insight into the pathophysiology of T2D. This highlights the role of FFAR3 as a possible target for future anti-diabetic drugs and treatments.

## Highlights

• In small intestinal, FFAR3 expression in enteroendocrine cells is markedly influenced by diet, especially high fat diet.
• The number of enteroendocrine cells is affected by diet in normal weight individuals but not in the subjects with obesity.
• Intestinal produced ketone bodies inhibit GLP-1 secretion in enteroendocrine cells via FFAR3 and Gα_i/o_ signaling pathway.

## Graphical abstract

**Figure:**
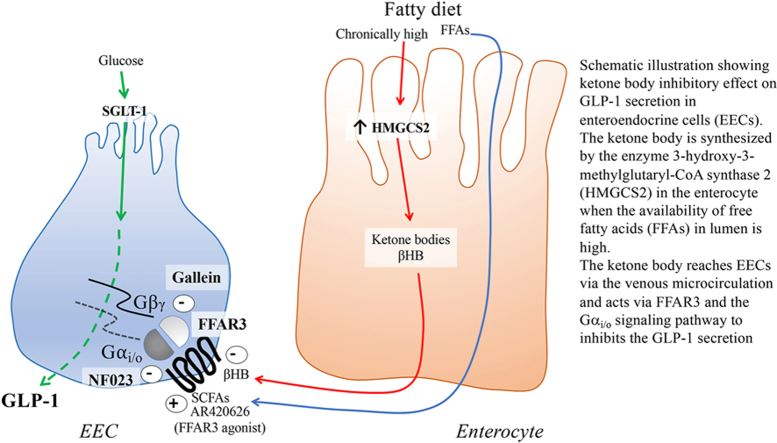

## 1 Introduction

Glucagon-like peptide 1 (GLP-1) is becoming an increasingly utilized target for treating obesity and associated conditions such as type 2 diabetes mellitus (T2D). This incretin hormone is a key player in glucose- and energy-homeostasis. Released from enteroendocrine L-cells in the small intestine in response to stimulation by nutrients, it is a satiety signaling hormone in the hypothalamus and has glucose balancing effects on β-cells such as increased glucose sensitivity and insulin secretion as well as increased cell proliferation [1]. GLP-1 also increases insulin sensitivity in peripheral tissues [2]. Enteroendocrine cells (EECs) can be triggered to release GLP-1 by both glucose and short-chain fatty acids (SCFAs) [3]. Glucose stimulates GLP-1 through the Sodium-Glucose Transporter (SGLT)-1 and SCFAs such as acetate, propionate and butyrate through free fatty acid receptors (FFARs) which are G-protein coupled receptors (GPCRs) [[4], [5], [6], [7], [8], [9]]. Gut microbiota produce SCFAs via dietary fermentation of fiber, which then can regulate energy homeostasis in EECs via FFARs, such as FFAR2 and FFAR3, located apically in the cells. However, FFARs in general are expressed not only at the apical luminal tip of EECs but also at the basolateral membrane [8]. Thus, one function of FFARs at the basolateral membrane of EECs, primarily in the small intestine, may be to sense the breakdown metabolites from absorbed nutrients, which have been released from enterocytes into the microvillar blood circulation and reach the EECs at the basolateral side.

It has been shown that GLP-1 secretion is blunted in obesity and T2D, which has been suggested to contribute to the decreased insulin secretion and increased insulin resistance seen in these conditions [10]. A study by our group recently identified high expression of the ketogenesis rate-limiting enzyme mitochondrial 3-hydroxy-3-methylglutaryl-CoA synthase 2 (HMGCS2) in the jejunum of obese patients prior to Roux-en-Y gastric bypass (RYGB) surgery and a substantial decrease of this enzyme after surgery [11,12]. HMGCS2 is the rate-limiting enzyme for the production of ketone bodies like β-hydroxybutyrate (βHB) in the jejunal enterocytes when the substrate SCFAs for ketogenesis are available [11]. We have shown that these ketone bodies have an inhibitory effect on GLP-1 secretion from EECs via an hitherto unidentified GPCR [8]. After RYGB surgery, bile salts and fat-dissolving pancreatic enzymes are mixed with food further down the small intestine at the jejuno-jejunostomy and triglycerides can no longer be digested in the alimentary limb because of absence of gastric and pancreatic lipases, leading to the cessation of ketone production in the proximal 1.5–2 m of the jejunum [13]. We suggested that this may contribute to the increased GLP-1 levels after RYGB and have an immediate effect on the improved glucose homeostasis and improvement of T2D in the immediate phase after RYGB surgery before any weight loss has occurred [12].

The ketone bodies participate in various cellular processes as signaling molecules. Recent study has shown that the ketone body βHB activates physiological functions via the FFAR3 [14]. GPCRs, like FFAR3, are activated by ligands where the α-subunit coupled to the receptor disassociate from the βγ-subunits and further affect intracellular downstream signaling [15]. When not stimulated by a receptor, Gα is bound to Gβγ to form the inactive G-protein trimer. FFAR3 has been described to activate the Gα_i/o_-subunit pathways [4,8]. We have shown that FFAR3 expression is increased by a high-fat diet (HFD) in the upper gut in healthy non-obese individuals [16]. We therefore wanted to investigate the role of FFAR3 in GLP-1 secretion from EEC to further explore the mechanisms behind the rapid T2D remission after bariatric surgery. The aim of the study was to evaluate FFAR3 expression in EECs of the small intestine under different circumstances, such as diet and bariatric surgery. GLUTag cells were used to study FFAR3 signaling pathways.

## 2 Materials and methods

### 2.1 Ethics

Written consent from all study participants were granted in accordance with the Declaration of Helsinki. Ethics were approved by the Regional Ethical Review Board in Gothenburg, Sweden (Dnr: 007–09, 807–11, and 001–11) and the Ethics Committee of Gothenburg University. Ethics for murine studies were approved by the Gothenburg Committee of the Swedish Animal Welfare Agency (Dnr: 246–2009).

### 2.2 Enteroscopy

Endoscopy was performed to collect human biopsies from the duodenal and jejunal mucosa approximately 50 cm distal to the ligament of Treitz. Subjects received sedation with Midazolam and Alfentanil prior to endoscopy. Two to three biopsies from each level were snap frozen in liquid nitrogen or chemically fixed for later analysis.

### 2.3 Human samples

1. Samples from healthy normal weight controls were taken from the duodenum and the jejunum. The group consisted of 11 male individuals with a mean age of 25.7 years (range 21–30 years) and a BMI average of 23 kg/m^2^ (range 18.6–28.4).
2. Samples from jejunum were also taken from 15 healthy normal weight individuals before and after diet intervention with two diets in a cross-over fashion. In between the diets there was a wash-out period of two weeks. The group consisted of 8 men and 7 women with a mean age of 25.5 years and a BMI average of 23 kg/m^2^ (range 18.7–25.1). A high fat diet (HFD) and a high carbohydrate diet (HCD) were each followed for 2 weeks. The energy content for the diets were 60 % fat and 60 % carbohydrates, respectively. The participants were instructed not to eat anything but the food and drinks provided by the laboratory, but were free to drink extra tap water if needed. The study was registered at ClinicalTrials.gov (NCT02088853) and was performed at Dept. of Surgery, Sahlgrenska University hospital, Gothenburg between February and December 2014. Three reports have already been published regarding this study; first a report on the systemic glucose clearance following a mixed meal test [17], a second report on glucose transport in jejunal biopsies [16] and a third morphological report on mucosal adaptation in relation to the dietary intervention [18].
3. Biopsy material from the duodenal and jejunal mucosa were collected from 12 obese patients prior to a very low-energy diet (VLED) and RYGB surgery, from the jejunum during surgery and 6–8 months after surgery. Collection sites were the same before and after surgery. Half of the patients were diagnosed with T2D prior to surgery according to WHO guidelines for HbA1c. The group consisted of 3 men and 9 women and the mean age among the group was 52.8 years (range 40–63 years) and the mean BMI 39.6 kg/m^2^ (range 37.1–42.5).

### 2.4 Animal samples

Murine samples were taken from male C57BL/6J mice (Harlan, Horst, The Netherlands) who underwent either a HFD diet (Surwit Diabetogenic Rodent Diet, D12309; Research Diets, New Brunswick, New Jersey, USA) or low-fat normal chow (NC) (R34, Lactamin, Kimstad, Sweden) for 3 months from the age of 6–8 weeks. Prior to this, they received ad libitum NC with a nutrient content of 4 % fat, 16.5 % protein and 58 % carbohydrate. HFD had a nutrient content of 35.9 % fat, 23 % protein and 35.5 % carbohydrate. The NC diet mice continued to eat the previous diet (R34). The mice were sacrificed after 3 months and samples were taken from the small intestine.

### 2.5 GLUTag cell cultures

Murine GLUTag enteroendocrine intestinal cells is an established cell line produced by Dr. Daniel Drucker. Thawed cells were incubated in Dulbecco's Modified Eagle (DMEM, Low glucose 5 mM, Invitrogen, Thermo Fisher Scientific) medium at 37 °C for two days and later detached using Trypsin EDTA (Thermo Fisher Scientific) for 5 min. Cells were centrifugated at 2000 rpm for 5 min and diluted in DMEM. Divided cells were either added to 24-well plates and incubated for three days or saved for future experiments. Saline secretion buffer (138 mM NaCl, 4.5 mM KCl, 4.2 mM NaHCO_3_, 1.2 mMNaH_2_PO_4_, 2.6 mM CaCl_2_, 1.2 mM MGCl_2_, 10 mM HEPES and 0.1 % BSA, pH 7.4) was used for preincubation for 1 h at 37 °C. The cells received glucose 0.5 mM, in combination with either 10–100 μM AR420626 (FFAR3 agonist, Sigma-Aldrich, Darmstadt, Germany), 10–100 μM NF023 (Gα_i/o_-specific antagonist, Sigma-Aldrich), 10–100 μM Gallein (inhibitor of Gβ/γ signaling, Sigma-Aldrich) and/or 100 mM β-hydroxybutyrate (Sigma-Aldrich). After this, cells were stimulated for 2 h with either 1 mM glucose or in combination with the agents used for pre-incubation. Dipeptidyl peptidase-4 inhibitor (Sigma-Aldrich) was added as the last step before saving supernatant for enzyme-linked immunosorbent assay (ELISA). The cells were scraped off in each well and saved for Western blot analysis. GLP-1 secretion is related to the protein concentration (to avoid treatment affecting the cell number), as well as to each experiment's control trial.

### 2.6 GLP-1 quantification

ELISA assay was performed following instructions for GLP-1 Total ELISA kit (EMD Millipore Corporation, St. Louis, Missouri, USA). GLP-1 was quantified in GLUTag cell culture media samples in relation to GLP-1 standard which is read at absorbance 450 and 590 nm.

### 2.7 Western blot

Protein concentration in samples collected from enteroscopy and GLUTag experiments were analyzed using Bradford protein assay and Bovine serum albumin (Bio Rad Laboratories, Hercules, California, USA) was used for standard curve. First, RIPA buffer (Sigma-Aldrich) containing Complete TM Protease inhibitor cocktail (Bio Rad Laboratories) was added to the samples before sonication and centrifugation for 10 min at 4 °C at 12000G. Bio-Rad Protein Assay Dye Reagent Concentrate (Bio Rad Laboratories) was added to each sample and absorbance was read at 590 nm in a 96-well plate. Calculated samples were incubated at 95 °C for 5 min in Laemmli Sample Buffer and β-mercaptoethanol (Bio Rad Laboratories) and added to Bio-Rad Criterion™ TGX Stain-free™ 4–15 % gel (Bio Rad Laboratories). For size comparison we used i-Bright™ protein prestained ladder (Thermo Fisher Scientific, Waltham, Massachusetts, USA) and All Blue (Bio Rad Laboratories) ladder. Chemi-doc™ MP Imager (Bio Rad Laboratories) was used for gel activation and transfer confirmation after transferring of proteins to a polyvinylidene difluoride membrane using Bio-Rad's Trans-Blot Turbo with high molecular weight settings.

Membranes were washed in wash buffer (Phosphate-buffered saline (PBS), 1 % Tween 20 (Merck KGaA, Darmstadt, Germany)) and block buffer (PBS, 2 g I-Block (Thermo Fisher Scientific)). Primary antibody FFAR3 (PA5-75521, ThermoFisher Scientific, dilution 1:500) was used for nightly incubation at 4 °C. Horseradish peroxidase (HRP)-linked secondary antibody (7074S, Polyclonal Anti-rabbit IgG Secondary Antibody, Cell Signaling Technology, Danvers, Massachusetts, USA, dilution 1:2000) was used for incubation at 1 h in room temperature. Chemiluminescent Clarity Max Western ECL (Bio Rad Laboratories) was used to visualize proteins in the Chemi-doc™ MP Imager using a stain-free normalization method.

### 2.8 Immunohistochemistry

The chemically fixed samples were prepared and embedded in paraffin. The cut slices were washed in Tissue Clear (Histolab Products AB, Askim, Sweden) followed by 99.5 % ethanol, 95 % ethanol and PBS. After this, the cut slices were boiled in 10 mM citrate buffer (pH 6.0) for 20 min to retrieve antigens, before blocking in 5 % normal goat serum. Slices were incubated overnight in the primary antibodies Chromogranin A (Human: Ab15160, Abcam, Cambridge, Great Britain. Mouse: Thermo Fisher Scientific, PA5-77917. Dilution 1:100) and FFAR3 (PA5-75521, dilution 1:100, ThermoFisher Scientific) at 4 °C. Some slides were double stained. The morning after, the slices were washed and incubated in darkness at room temperature with the secondary antibodies (AlexaFluor 488 goat anti-rabbit IgG, cat. No: A11008, dilution 1:1000, Thermo Fisher Scientific and AlexaFluor 568 goat anti-mouse IgG, cat. No: A11031, dilution 1:1000, ThermoFisherScientific) for 1.5 h. After additional washing, slices were counter-stained using Hoechst staining and cover-slipped with ProLong Gold anti-fade agent (Thermo Fisher Scientific). Blocking buffer was used as negative control. Slides were analyzed using a fluorescence microscope (Leica Microsystems, Wetzlar, Germany). The numbers of EECs in the jejunal mucosa were blindly reviewed by four researchers independent of each other. An estimate of the mucosa size was made with a grid, where the number of EECs and points of intersection were counted. Calculations are based on the number of EECs in relation to number of points of intersection in each individual sample.

### 2.9 Statistical analysis

GraphPad Prism 9 was used for data analysis. Because the normal distribution is unknown, samples were analyzed using nonparametric Wilcoxon signed-rank test for Paired groups (human subjects) while nonparametric Mann-Whitney was used to analyze unpaired groups (mice). Friedman test was used to compare interventions in subjects with obesity. Cell cultures were analyzed using one-way ANOVA with Dunnett's multiple comparison test when comparing multiple unpaired groups with one variable. Statistical significance is defined as a p-value <0.05.

## 3 Results

### 3.1 FFAR3 expression in normal weight controls and individuals with obesity undergoing RYGB

**Fig. 1:**
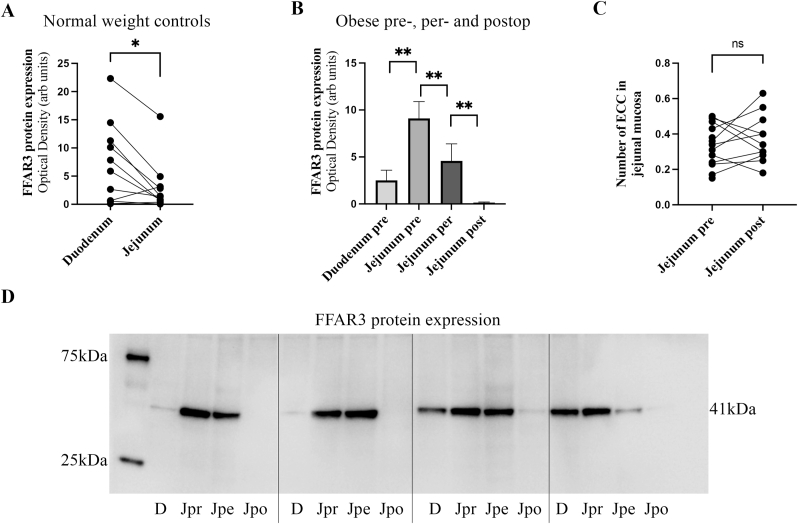
In **A**, Western blot analysis of Free fatty acid receptor 3 (FFAR3) in duodenum and jejunum in normal weight controls (n = 11). In **B**, Western blot analysis of FFAR3 in duodenum and jejunum pre very low-energy diet (VLED), in jejunum after VLED per-Roux-en-Y gastric bypass (RYGB) and in jejunum 6–8 months post-RYGB in obese patients (n = 12). In **C**, the number of enteroendocrine cells (EEC) is calculated in tissue sections stained with chromogranin A in jejunum pre- and post-RYGB in obese patients (n = 10). In **D,** representative samples of protein bands of FFAR3 in obese patients. The membrane is showing 4-paired individuals with typical optical density. Statistical analyses were performed using Friedman test where appropriate followed by the Wilcoxon signed-rank test for paired samples. *p < 0.05, **p < 0.01. D, duodenum; Jpr, jejunum pre-RYGB; Jpe, jejunum per-RYGB; Jpo, jejunum post-RYGB.

Protein expression of FFAR3 was compared intraindividually between the duodenum and jejunum in normal weight human subjects. The expression of FFAR3 was lower in the jejunum compared to the duodenum (p = 0.00195, Fig. 1A). Individuals with obesity were compared intraindividually pre-, per- and post-RYGB surgery. Six of the twelve individuals had T2D before RYGB but 6–8 months after surgery all were in remission according to HbA1c levels (mean BMI 30.7 ± 0.7 kg/m^2^, range 27–35.8 kg/m^2^, mean HbA1c 38.5 ± 4 mmol/mol). In the subjects with obesity, before surgery, the jejunal protein expression of FFAR3 was significantly higher in the jejunum compared to the duodenum (p = 0.001, Fig. 1B). After the preoperative VLED the perioperative jejunal FFAR3 was significantly decreased (p = 0.001, Fig. 1B). Six to eight months after RYGB surgery jejunal FFAR3 expression was even further decreased (p = 0.001, Fig. 1B). The expression pattern for patients with T2D did not differ compared those without (data not shown). The number of EECs was calculated in tissue sections stained with chromogranin A, a marker of EECs, in the jejunum pre- and post-RYGB in subjects with obesity. The number of EECs remained unchanged pre- and post-RYGB (p = ns, Fig. 1C). These data indicate that the expression of FFAR3 is affected both by dietary changes and by the surgical re-routing which leads to a lack of free fatty acids for binding to FFAR3. A representative Western blot membrane shows the protein bands of FFAR3 in four obese patients in duodenum and jejunum pre- peri- and post-RYGB (Fig. 1D).

**Fig. 2:**
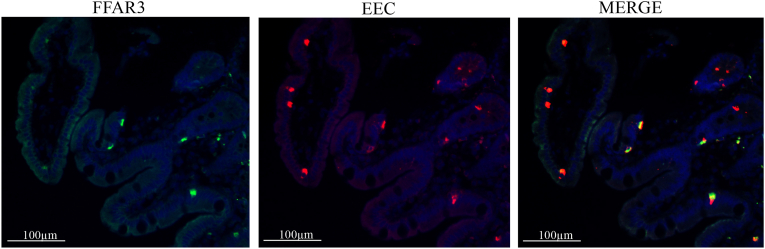
Representative double stain immunofluorescence of Free fatty acid receptor 3 (FFAR3) and enteroendocrine cells (EEC) marker chromogranin A in jejunum in one obese patient. Staining for FFAR3 are shown green, EEC are shown red, colocalization (merge) are shown yellow and blue staining are shown nucleus. (For interpretation of the references to colour in this figure legend, the reader is referred to the Web version of this article.)

Using double immunofluorescence staining with FFAR3 and chromogranin A antibody we found that the FFAR3 receptor was localized to some EECs that are likely to represent L-cells (Fig. 2). Since the number of EECs was unchanged before compared to after RYGB, the proportion of FFAR3 positive EECs was greatly reduced after surgery. This means that the number of EECs is not significantly affected by either diet or surgery in obese patients, while FFAR3 expression decreases or completely disappears in some EECs depending on diet and RYGB surgery which reduces the availability of the substrate free fatty acids.

### 3.2 FFAR3 and EECs increase after high-fat diet

**Fig. 3:**
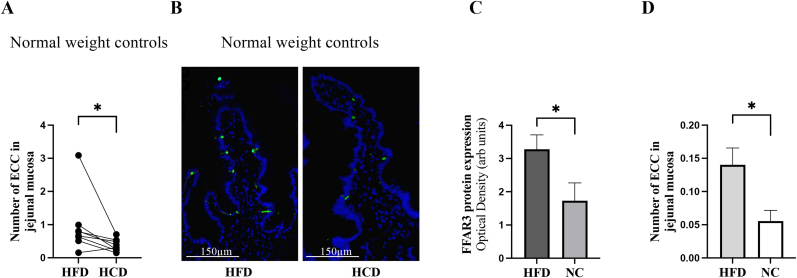
In **A**, the number of enteroendocrine cells (EEC) is calculated in tissue sections stained with chromogranin A in normal weight controls after a diet high in fat (HFD) or high in carbohydrate (HCD) (cross-over design) (n = 8, paired samples). In **B**, representative immunofluorescence staining of EECs marker chromogranin A (green) and nucleus (blue) in one normal weight individual. In **C**, Western blot analysis of Free fatty acid receptor 3 (FFAR3) in mouse small intestinal after HFD (n = 6) or normal chou (NC) (n = 6). In **D**, the number of EECs is calculated in tissue section from mouse small intestinal after HFD or NC using the marker chromogranin A. Statistical analysis were performed using Wilcoxon signed-rank test for paired samples or Mann-Whitney test for unpaired groups (mice). *p < 0.05. (For interpretation of the references to colour in this figure legend, the reader is referred to the Web version of this article.)

Previously published results from our group showed that FFAR3 protein expression (but not FFAR2) was significantly increased by a two-week HFD period compared to HCD in fifteen healthy non-obese individuals [16]. In the present study, we show in the same population that the number of EECs increased after HFD (p = 0.0156, Fig. 3A). A limitation, however, is that the number of individuals is slightly fewer when calculating EECs (n = 8) and that there is a greater effect of one participant compared to the others. Nevertheless, the difference in the number of EECs remains even if this person is excluded. In Fig. 3B a representative staining with chromogranin A in one healthy individual shows the distribution of EECs after two-weeks of HFD followed by two-weeks of HCD (Fig. 3B). It is therefore possible that the increased expression of FFAR3 is mainly due to the increased number of EECs in the jejunal mucosa (Fig. 3B).

To validate the human data, mouse experiments were performed. Using Western blot analysis, the expression of FFAR3 was increased in murine small intestinal mucosa after three months of HFD compared to NC (p = 0.0159, Fig. 3C). Just as in the normal weight humans, the number of EECs increased on the HFD compared to NC fed animals (p = 0.02, Fig. 3D). The mouse experiments corroborate that FFAR3 expression may vary mainly with the number of EECs.

### 3.3 FFAR3 agonist stimulates GLP-1 secretion

**Fig. 4:**
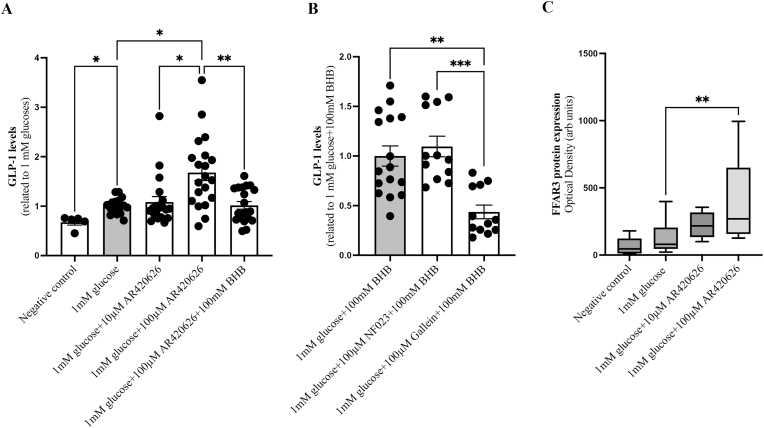
In **A**, Glucose-like peptide-1 (GLP-1) levels in cell culture media from GLUTag cells after incubation with 0 mM glucose (negative control), 1 mM glucose, and 1 mM glucose in combination with 10 or 100 μM free fatty acid receptor 3 (FFAR3) agonist AR420626 (n = 20) and/or the 100 mM ketone body β-hydroxybutyrate (βHB) (n = 20). In **B**, GLP-1 levels in culture medium from GLUTag cells after incubation with 1 mM glucose and 100 mM βHB (n = 16) in combination with 100 μM Gα_i/o_-specific antagonist NF023 (n = 12) or 100 μM Gallein, an inhibitor of Gβ/γ signaling pathway (n = 12). In **C**, Western blot analysis of FFAR3 in GLUTag cells incubated with FFAR3 agonist AR420626 (n = 8–12). Data has been related to 1 mM glucose or 1 mM glucose in combination to 100 mM βHB. Statistical analyses were performed using one-way ANOVA with Dunnett's multiple comparisons test. *p < 0.05, **p < 0.01, and ***p < 0.001.

Individual cell culture experiments showed different absolute values of GLP-1 secretion, measured in the cell culture medium using ELISA, and were therefore normalized to controls (as described above) in order to be comparable between experiments. GLUTag cells showed an increased GLP-1 secretion after stimulation with 1 mM glucose compared to vehicle (p = 0.0019, Fig. 4A). The FFAR3-specific agonist AR420626 at the higher dose of 100 μM further increased GLP-1 secretion from GLUTag cells compared to 1 mM glucose only (p = 0.0288, Fig. 4A). When treated with the FFAR3 agonist AR420626 in combination with the ketone body βHB, GLP-1 secretion was decreased (p = 0.0088, Fig. 4A). This shows that the ketone body βHB has the ability to block GLP-1 secretion via the FFAR3 receptor. To study which signaling mechanism βHB acts on, the GLUTag cells were treated with a combination of the ketone body βHB and a specific Gα_i/o_-signaling antagonist or a Gβ/γ inhibitor.

There was no significant inhibitory effect of GLP-1 secretion in GLUTag cells receiving the Gα_i/o_-specific antagonist NF023 in combination of the ketone body βHB, compared to the ketone body βHB alone (p = ns, Fig. 4B). However, cells treated with a combination of the ketone body βHB and Gallein, an inhibitor of Gβ/γ signaling, displayed significantly decreased GLP-1 secretion (p = 0.0025, Fig. 4B). This suggests that the ketone body βHB does *not* block this signaling pathway but rather exerts its inhibitory action on GLP-1 secretion via the Gα_i/o_-signaling pathway.

After the cell experiments, the protein expression of FFAR3 in the GLUTag cells was measured using Western blot analysis. There was a significant increase in FFAR3 expression in cells treated with the FFAR3-specific agonist AR420626 at the higher dose of 100 μM compared to 1 mM glucose (p = 0096, Fig. 4C).

**Fig. 5:**
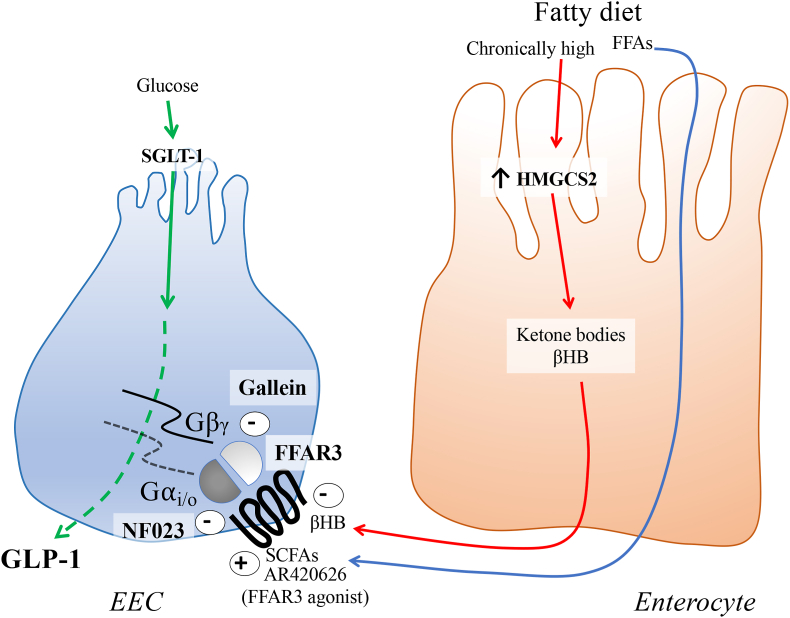
Schematic illustration showing ketone body inhibitory effect on GLP-1 secretion in enteroendocrine cells (EECs). The ketone body is synthesized by the enzyme 3-hydroxy-3-methylglutaryl-CoA synthase 2 (HMGCS2) in the enterocyte when the availability of free fatty acids (FFA) in lumen is high. The ketone body reaches EECs via the venous microcirculation and acts via FFAR3 and the Gα_i/o_ signaling pathway to inhibits the GLP-1 secretion.

Fig. 5 shows a schematic presentation of the regulation of GLP-1 secretion in EECs via FFAR3 and βHB produced by adjacent enterocytes and the FFAR3 signaling pathways Gα_i/o_ and Gβ/γ.

## 4 Discussion

In previous studies, we have shown that GLP-1 secretion from the jejunum is blunted in subjects with obesity and a part of the explanation behind this may be intestinal ketogenesis, induced by e. g. HFD, where high expression of HMGCS2 produces ketone bodies in the enterocytes that inhibit GLP-1 secretion from adjacent EECs. In the present study using GLUTag cells, we now show that GLP-1 secretion is inhibited by the ketone body βHB via the GPCR FFAR3 and via the Gα_i/o_ signaling pathway. Further, the expression of FFAR3 seems to be increased by HFD. The number of EECs was changed dependent on diet in healthy individuals but not in the subjects with obesity regardless of the diet.

There was a significant difference in the FFAR3 expression in the duodenum compared to the jejunum in healthy normal weight subjects compared to subjects with obesity with and without T2D. In the normal weight subjects, the FFAR3 expression was higher in the duodenum while the opposite was seen in subjects with obesity. This has not been shown in previous studies. Preoperative VLED decreased FFAR3 expression in the subjects with obesity. While there is no standard for what constitutes a VLED, it often has a higher protein content and lower carbohydrate and fat content than the average diet. The changes in FFAR3 after VLED could be caused by the composition of the diet, in particular the low-fat content but that cannot be concluded from the present study. After RYGB surgery, the expression of FFAR3 was nearly abolished in the alimentary limb in both obese subjects with and without diabetes. However, the number of EECs did not seem to change from the pre-surgery VLED period of two weeks to post-RYGB. With the help of double staining immunofluorescence, we found that FFAR3 was expressed in a subset of EECs as previously described [8]. We also found that a HFD compared to HCD induced a higher jejunal FFAR3 expression and EEC numbers in both normal weight humans and mice [16]. This suggests that FFAR3 expression and the number and density of EECs are linked and both are affected by the diet. In Elebring et al. (2022), an increase in HMGCS2 expression and postprandial GLP-1 secretion was seen after a two-week HFD in normal weight humans [17]. Since the L-cells specifically are responsible for GLP-1 secretion [3], the increase in numbers of these cells can explain the increase in GLP-1 levels post HFD in the normal weight subjects. This increase in the EEC number after two-week HFD could be a sort of short-term compensational mechanism to allow the small intestine to respond adequately to the amount of fat consumed in the diet [19,20]. We suggest that when more fat (fatty acids) that is consumed, it induces increased numbers of EECs and expression of FFAR3. The net effect of this in healthy individuals is increased GLP-1 secretion despite the simultaneous increase in HMGCS2 leading to increased jejunal ketone body production.

We expected that the number of EECs would change according to diet also in the subjects with obesity based on the results seen after HFD and HCD in normal weight subjects. What we actually observed was that the number of EECs remained unchanged. We speculate that the mucosa of subjects with obesity does not adapt adequately to the dietary fat content compared to in healthy individuals. Increased fat intake leads to induction of HMGCS2 and increased levels of ketone bodies in the jejunal mucosa. The number of EECs however remains stationary, which allows the ketone bodies to inhibit the L-cells and subsequently decreases GLP-1 secretion pre-RYGB. The reasons behind this lack of adaptation cannot be concluded from the present study, it is likely multifactorial – diet and weight gain by itself could contribute, but it could also be caused by genetic variability. It might also be caused by other long-term changes in subjects with obesity, e. g. epigenetics. This contradicts previous knowledge of the EEC density in different parts of the gastrointestinal tract and could illustrate that mucosal histomorphological changes could be part of the pathogenesis of obesity and T2D [21,22]. After RYGB, however, we have previously shown that HMGCS2 expression and ketone body levels in the small intestine decrease significantly, contributing to decreased inhibition of the L-cells and thereby normalized GLP-1 secretion [11]. Since we have not yet studied how subjects with obesity respond to HFD compared to HCD, it is of interest to study this in order to test this hypothesis.

Previous studies on mouse small intestinal cultures have shown that the ketone body βHB inhibits GLP-1 secretion via a GPCR-mediated mechanism using the Gα (i/o) inhibitor pertussis toxin [11]. We have also shown that ketone body βHB inhibits GLP-1 secretion in GLUTag cells, which is an enteroendocrine cell line [23]. The doses used in the present study on the GLUTag cells study may be considered high but can be explained by the high density of EECs in these *in vitro* experiments compared to the *in vivo* situation [23]. In the present study we show that GLP-1 secretion from GLUTag cells was triggered by the FFAR3 specific agonist AR420626. The synthetic molecule AR420626 is an allosteric ligand to FFAR3 and has highlighted the role of this receptor in GLP-1 release [24]. The action of the ketone body βHB on FFAR3 still needs to be concluded, i. e. whether it is a potential orthosteric agonist or an antagonist [24]. When we combined AR420626 with ketone body βHB the GLP-1 secretion decreased. As GLP-1 production was not completely blocked, it may be due to a competitive action between AR420626 and βHB. It is possible that βHB is either an antagonist or a less potent agonist compared to AR420626. When we examined the FFAR3 expression in the GLUTag cells after AR420626 stimulation, an interesting finding was discovered. The AR420626 agonist significantly increased FFAR3 expression. In our previous studies, we also found that the ketone body βHB increases FFAR3 expression [23]. This indicates that FFAR3 is rapidly activated which can affect the ability of AR420626 to exert its agonistic effects and counteract the inhibitory effect of βHB.

A study by Kaji I et al. (2018) showed that the effect of the FFAR3 agonist AR420626 was reversed in presence of the Gα (i/o) inhibitor pertussis toxin on nicotine-evoked contractions [25]. NF023 is a specific and direct G-protein antagonist that acts on α-subunits of G_i/o_ [14]. NF023 in combination with the ketone body βHB did not show any additional GLP-1 inhibition. Disregarding factors for NF023 such as possibly low receptor specificity, concentration, or sensitivity, the results indicate that the ketone body βHB exert its effect via this signaling pathway as it was not possible to block GLP-1 secretion more with NF023. Studies on the enteroendocrine cells NCI–H716 have shown that GLP-1 secretion also can be mediated via the Gβ/γ signaling pathway [26]. However, after treatment with Gallein, a Gβ/γ subunit signaling inhibitor, in combination with βHB, GLP-1 secretion decreased further. This suggests that the ketone body βHB is not capable of blocking this Gβ/γ signaling pathway. A schematic illustration of the action, i. e. the ketone body inhibitor action on FFAR3 is shown in Fig. 5.

In conclusion, the expression of FFAR3, colocalized to some EECs (possibly L-cells), is markedly influenced by diet, especially HFD, which increased FFAR3 protein expression in normal weight subjects. On the other hand, lack of substrate such as free fatty acids in the alimentary limb after RYGB, should downregulate FFAR3 expression. The number of EECs was affected by diet in the normal weight individuals but not in the subjects with obesity. This inability to compensate for the diet by the number of EECs, could be part of the pathogenesis of obesity and T2D. In GLUTag cells, we show that the ketone body βHB exerts its blocking effect on GLP-1 secretion via the FFAR3, and the Gα_i/o_ signaling pathway. Because FFAR3 is quickly regulated in response to both stimulatory and inhibitory agents, our study highlights the role of FFAR3 as a possible target for future anti-diabetic drugs and treatments.

## Funding

Stiftelsen Erik & Lily Philipsons Minnesfond 2019, 2020 (memorial fund), SLF student sommarforskningsstipendium 2021 (10.13039/501100007308Swedish Medical Association), Göteborgs läkaresällskap “Testa forskningsstipendium” 2021 (the Gothenburg society of medicine). The Sahlgrenska Academy (Amanuenstjänst 2022).

## CRediT authorship contribution statement

**Sara MT. Persson:** Writing – original draft, Investigation, Conceptualization. **Anna Casselbrant:** Writing – review & editing, Investigation, Conceptualization. **Aiham Alarai:** Writing – review & editing. **Erik Elebring:** Writing – review & editing, Investigation, Conceptualization. **Lars Fändriks:** Writing – review & editing. **Ville Wallenius:** Writing – review & editing, Conceptualization.

## Declaration of competing interest

None.

## References

[1] Marathe C.S., Rayner C.K., Jones K.L., Horowitz M. (2013). Glucagon-like peptides 1 and 2 in health and disease: a review. Peptides.

[2] Williams D.L. (2009). Minireview: finding the sweet spot: peripheral versus central glucagon-like peptide 1 action in feeding and glucose homeostasis. Endocrinology.

[3] Lim G.E., Brubaker P.L. (2006). Glucagon-like peptide 1 secretion by the L-cell: the view from within. Diabetes.

[4] Le Poul E., Loison C., Struyf S., Springael J.-Y., Lannoy V., Decobecq M.-E. (2003). Functional characterization of human receptors for short chain fatty acids and their role in polymorphonuclear cell activation. J. Biol. Chem..

[5] Rayasam G.V., Tulasi V.K., Davis J.A., Bansal V.S. (2007). Fatty acid receptors as new therapeutic targets for diabetes. Expert Opin. Ther. Targets.

[6] Ichimura A., Hirasawa A., Hara T., Tsujimoto G. (2009). Free fatty acid receptors act as nutrient sensors to regulate energy homeostasis. Prostag. Other Lipid Mediat..

[7] Tolhurst G., Heffron H., Lam Y.S., Parker H.E., Habib A.M., Diakogiannaki E., Cameron J., Grosse J., Reimann F., Gribble F.M. (2012). Short-chain fatty acids stimulate glucagon-like peptide-1 secretion via the G-protein-coupled receptor FFAR2. Diabetes.

[8] Lu V.B., Gribble F.M., Reimann F. (2021 Mar 9). Nutrient-induced cellular mechanisms of gut hormone secretion. Nutrients.

[9] Kuhre R.E., Deacon C.F., Holst J.J., Petersen N. (2021). What is an L-cell and how do we study the secretory mechanisms of the L-cell?. Front. Endocrinol..

[10] Faerch K., Torekov S.S., Vistisen D., Johansen N.B., Witte D.R., Jonsson A. (2015 Jul). GLP-1 response to oral glucose is reduced in prediabetes, screen-detected type 2 diabetes, and obesity and influenced by sex: the ADDITION-PRO study. Diabetes.

[11] Wallenius V., Elias E., Elebring E., Haisma B., Casselbrant A., Larraufie P. (2020). Suppression of enteroendocrine cell glucagon-like peptide (GLP)-1 release by fat-induced small intestinal ketogenesis: a mechanism targeted by Roux-en-Y gastric bypass surgery but not by preoperative very-low-calorie diet. Gut.

[12] Wallenius V., Dirinck E., Fändriks L., Maleckas A., le Roux C.W., Thorell A. (2018 Jun). Glycemic control after sleeve gastrectomy and roux-en-Y gastric bypass in obese subjects with type 2 diabetes mellitus. Obes. Surg..

[13] Pucci A., Batterham R.L. (2019 Feb). Mechanisms underlying the weight loss effects of RYGB and SG: similar, yet different. J. Endocrinol. Invest..

[14] Kimura I., Inoue D., Maeda T., Hara T., Ichimura A., Miyauchi S. (2011 May 10). Short-chain fatty acids and ketones directly regulate sympathetic nervous system via G protein-coupled receptor 41 (GPR41). Proc. Natl. Acad. Sci. U. S. A..

[15] Kimura I., Ichimura A., Ohue-Kitano R., Igarashi M. (2020). Free fatty acid receptors in health and disease. Physiol. Rev..

[16] Elebring E., Wallenius V., Casselbrant A., Docherty N.G., Roux C.W.L., Marschall H.U. (2022 May 7). A fatty diet induces a jejunal ketogenesis which inhibits local SGLT1-based glucose transport via an acetylation mechanism-results from a randomized cross-over study between iso-caloric high-fat versus high-carbohydrate diets in healthy volunteers. Nutrients.

[17] Wallenius V., Elebring E., Casselbrant A., Laurenius A., le Roux C.W., Docherty N.G., Biörserud C., Björnfot N., Engström M., Marschall H.U., Fändriks L. (2021). Glycemic control and metabolic adaptation in response to high-fat versus high-carbohydrate diets-data from a randomized cross-over study in healthy subjects. Nutrients.

[18] Casselbrant A., Wallenius V., Elebring E., Marschall H.U., Johansson B.R., Helander H.F., Fändriks L. (2022). Morphological adaptation in the jejunal mucosa after iso-caloric high-fat versus high-carbohydrate diets in healthy volunteers: data from a randomized crossover study. Nutrients.

[19] Gniuli D., Calcago A., Dalla Libera L., Calvani R., Leccesi L., Caristo M.E., Vettor R., Castagneto M., Ghirlanda G., Mingrone G. (2010). High-fat feeding stimulates endocrine, glucose-dependent insulinotropic polypeptide (GIP)-expression cell hyperplasia in the duodenum of Wistar rats. Diabetologia.

[20] Shackley M., Ma Y., tate E.W., Brown A.J.H., Frost G., Hanyaloglu A.C. (2020). Short chain fatty acids enhance expression and activity of the umami taste receptor in enteroendocrine cells via a Gα_i/o_ pathway. Front. Nutr..

[21] Osinski C., Le Gléau L., Poitou C., de Toro-Martin J., Genser L., Fradet M., Antoine Soula H., Leturque A., Blugeon C., Jourdren L., Ludiwyne Hubert E., Clément K., Serradas P., Ribeiro A. (2021). Type 2 diabetes is associated with impaired jejunal enteroendocrine GLP-1 cell lineage in human obesity. Int. J. Obes..

[22] Liu J., Zhang D., Yang Z., Hao Y., Wang Z., Wang J., Wang Z. (2023). Wheat alkylresorcinols modulate glucose homeostasis through improving GLP-1 secretion in high-fat-diet-induced obese mice. J. Agric. Food Chem..

[23] Elebring E., Casselbrant A., Persson S.M.T., Fändriks L., Wallenius V. (2023). βHB inhibits glucose-induced GLP-1 secretion in GLUTag and human jejunal enteroids. J. Mol. Endocrinol..

[24] Milligan G., Kimura I. (2017).

[25] Kaji I., Akiba Y., Furuyama T., Adelson D.W., Iwamoto K., Watanabe M., Kuwahara A., Kaunitz J.D. (2018). Free fatty acid receptor 3 activation suppresses neurogenic motility in rat proximal colon. Neuro Gastroenterol. Motil..

[26] Shin M.H., Choi E.K., Kim K.S., Kim K.H., Jang Y.P., Ahn K.S., Chung W.S., Cha N.H., Jang H.J. (2014). Hexane fractions of Bupleurum falcatum L. Stimulates glucagon-like peptide-1 secretion through Gβ/γ-mediated pathway. Evid Based Complement Alternat Med.

