# Supplementary figures and images for: Role of FFAR3 in ketone body regulated glucagon-like peptide 1 secretion
Source: Biochem Biophys Rep. 2024 Jun 7;39:101749. doi: 10.1016/j.bbrep.2024.101749 (PMC11192792; doi:10.1016/j.bbrep.2024.101749)

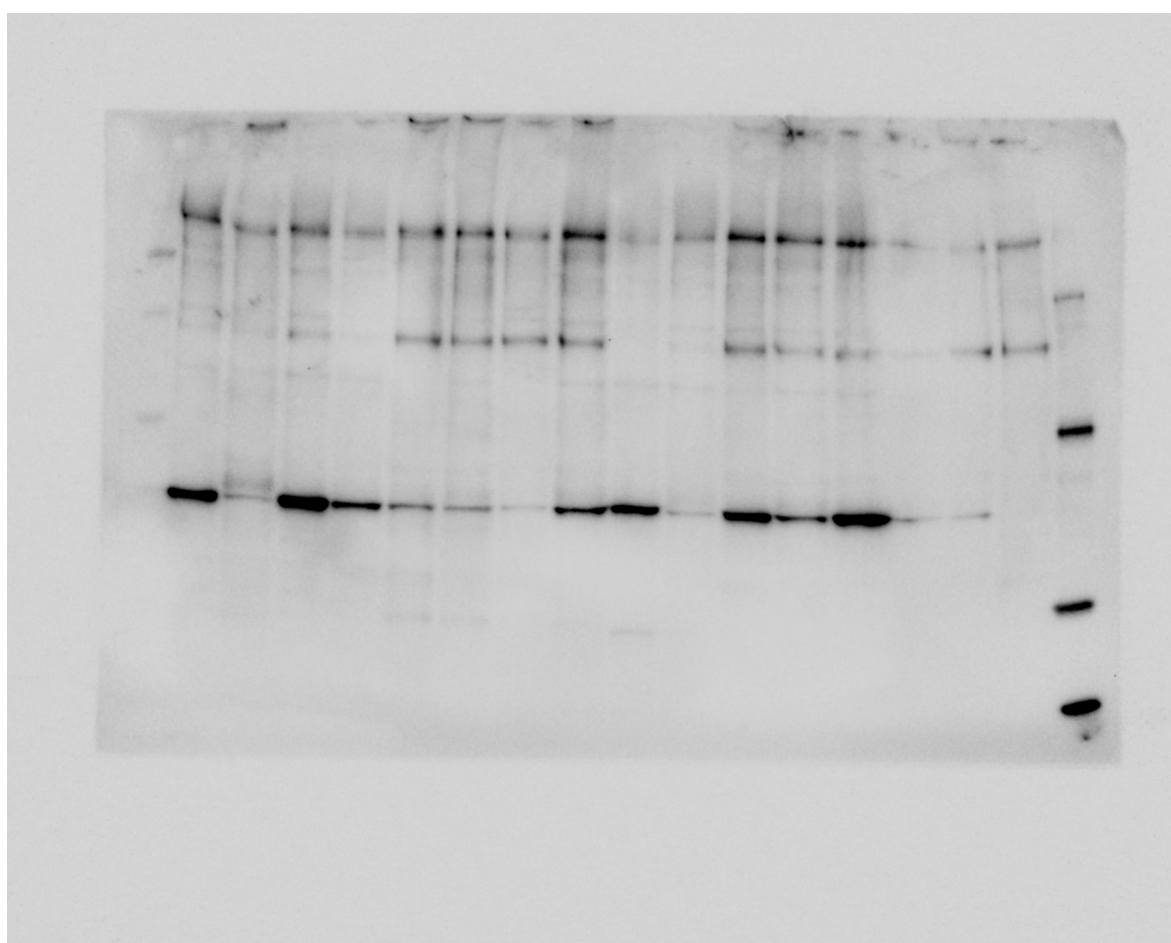

Supplement: Multimedia component 1 [file mmc1.pdf]

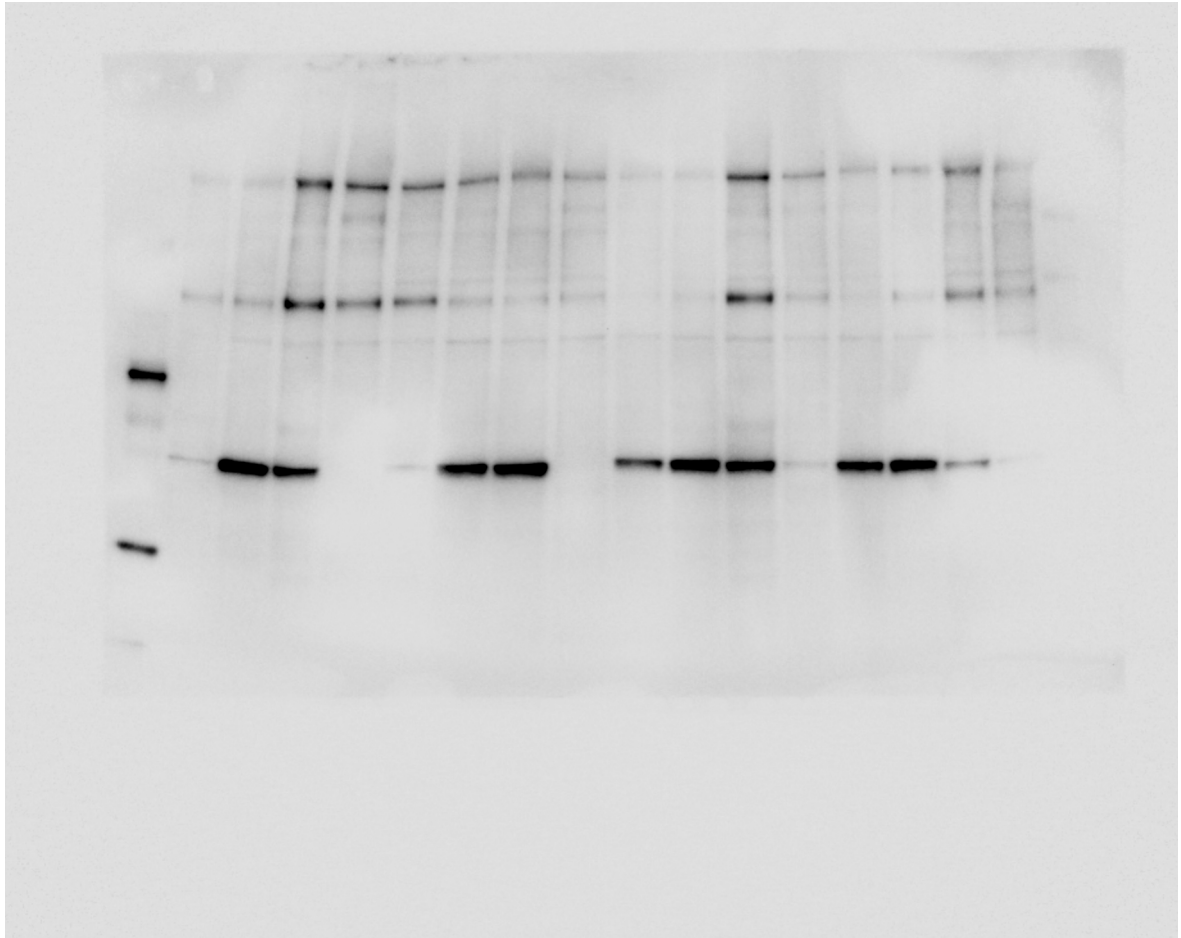

Supplement: Multimedia component 2 [file mmc2.pdf]

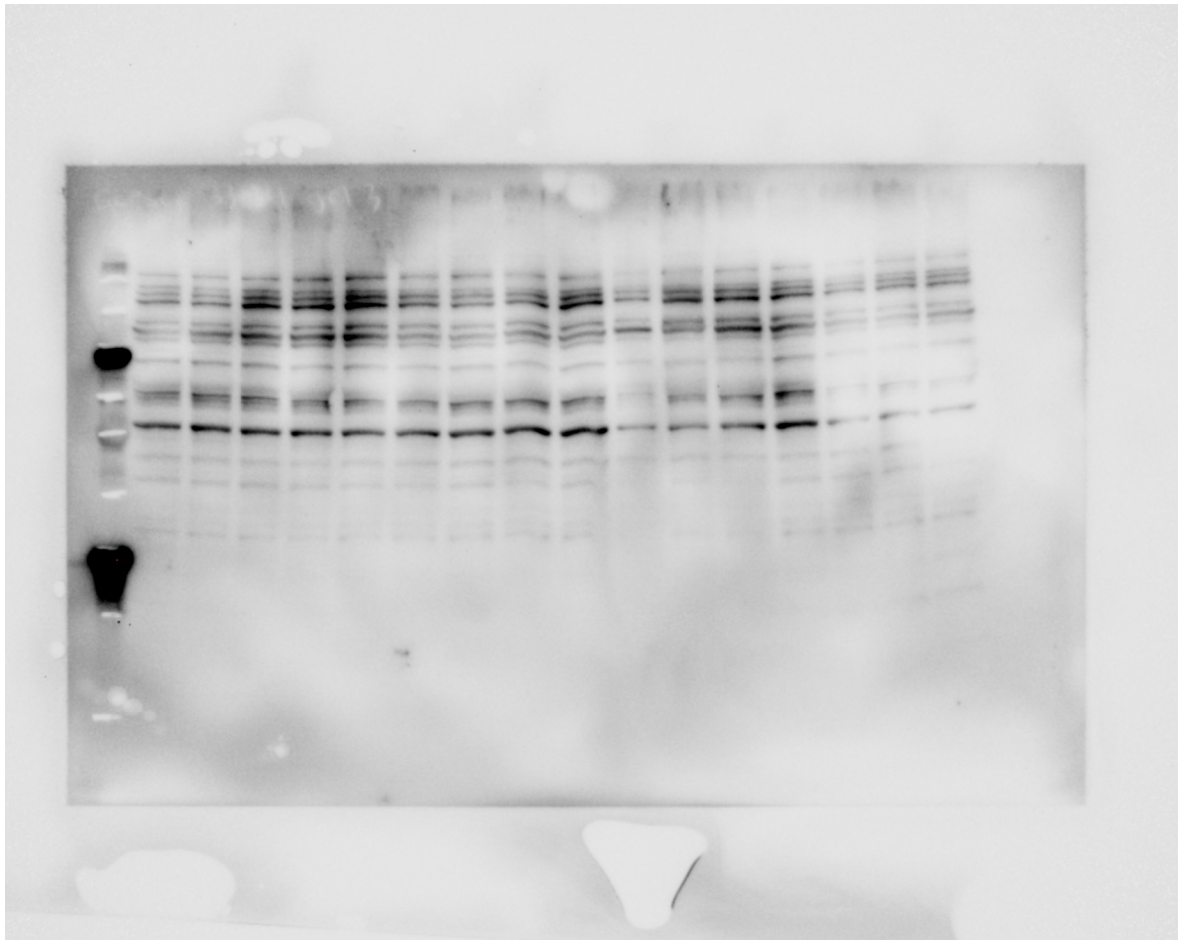

Supplement: Multimedia component 3 [file mmc3.pdf]

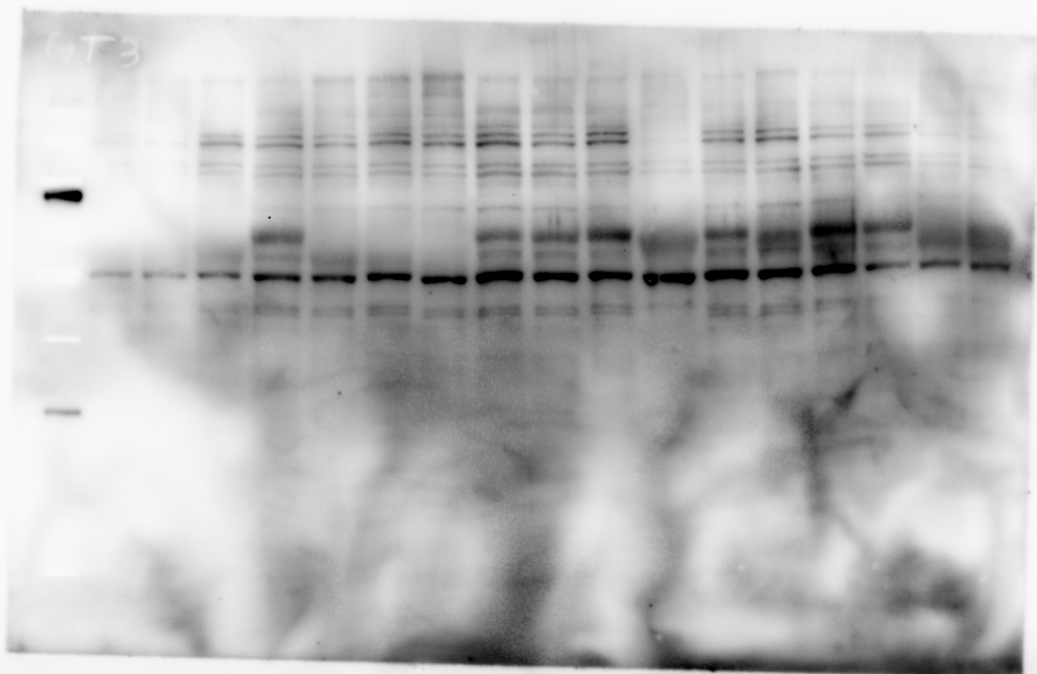

Supplement: Multimedia component 4 [file mmc4.pdf]
